# The domestication of South American camelids: a review

**DOI:** 10.1093/af/vfaa065

**Published:** 2021-06-19

**Authors:** Hugo D Yacobaccio

**Affiliations:** CONICET-Instituto de Arqueología, Universidad de Buenos Aires, Buenos Aires, Argentina

**Keywords:** South American camelid, domestication process, Mid-Holocene, Southern Andes

**Implications**

- Appearance of individuals larger than the current llama (earliest evidence around 7100 cal. BP, increasing between 5800 and 4200 cal. BP).
- Detection of human impact due to environmental management practices that suggests more intensive human intervention in the environment since ca. 5300–4869 cal. AP.
- Detection of pathologies indicative of human handling in bones of the extremities and vertebrae since ca. 4900 cal. AP.
- First appearance of corrals in caves or in stone structures in deep ravines on the edge of valleys between 4500 and 3639 cal. AP.

## Introduction

**Figure 1.**
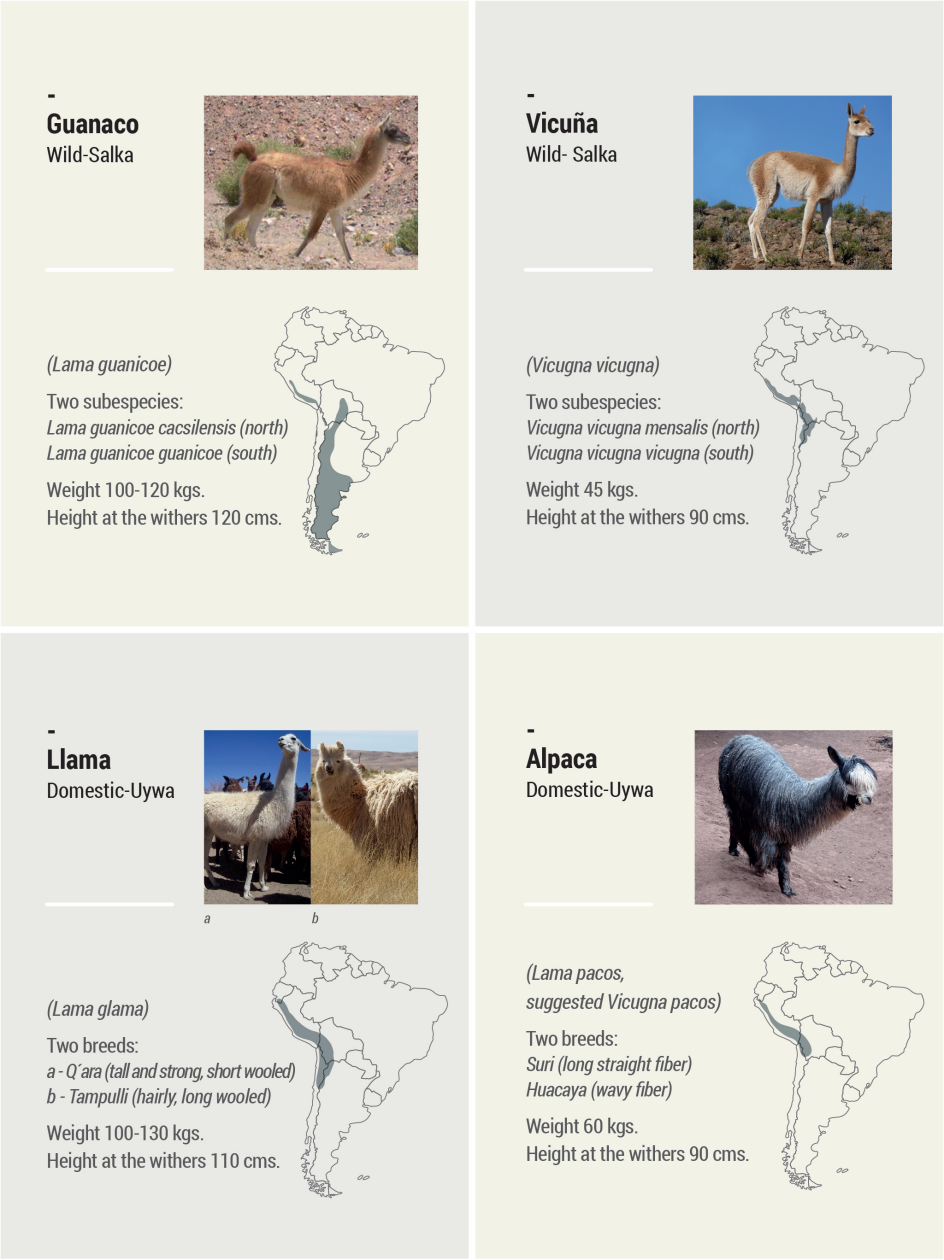
The four camelids: taxonomy, subspecies, breeds, and original distribution. Salka and Uywa are the Quechua names for wild and domestic, respectively.

South American camelids are the only domesticated ungulates in the Americas, and the Andean region sustained the only pastoralist societies in the pre-Hispanic New World. South American Camelids are composed by two genera and four species, two of them wild (vicuñas *Vicugna vicugna* Molina, 1782, and guanacos *Lama guanicoe* Müller, 1776) and two domestic (llamas *Lama glama* Linnaeus, 1758 and alpacas *Lama pacos* Cuvier, 1800 suggested *Vicugna pacos* Linnaeus, 1758 by Wheeler et al., 2006) (Figure 1).

Guanacos have a broad geographic distribution across a variety of open habitats (arid, semiarid, hilly, mountain, steppe) and temperate forest environments. Their social structure reflects this wide distribution, with some plasticity in types of groups. In the breeding season, the guanaco social structure comprises three basic units: territorial family groups, nonterritorial male groups, and solitary individuals. In turn, mixed groups are common during the winter and in some populations migration occurs. Family group territoriality in the breeding season is correlated with stable food supply (Vilá, 2012: 64). Vicuñas live only in high-altitude Puna environments above 3,400 m in Peru, Bolivia, Argentina, and Chile. They are adapted to open grasslands and steppes; although they prefer to graze in the humid wetlands or marshes (vegas), due to the presence of livestock in these wetlands, vicuñas are usually found in the steppes. Vicuñas live in family groups consisting of one male, three to four females, and two offspring, and in bachelor groups. Family groups are stable and territorial all year round (Vilá, 2012: 42–43).

During pre-Hispanic times, the domestic llamas were circumscribed to the Andean regions of Perú, Bolivia, Chile, and Argentina, but alpacas had a more restricted habitat in the high and humid punas (bofedales) of Perú, Bolivia, and northern Chile.

The domestication of camelids was a complex process associated with the adaptations of hunter-gatherer groups to environmental fragmentation, caused by increased aridity during the Mid-Holocene and the consequent loss of productive habitats in the region (Yacobaccio et al., 2017). During this period, hunter-gatherer groups adopted a logistic strategy, reducing their residential mobility and introducing technological innovations. They developed communal hunts of wild camelids, made possible by population aggregation during their annual cycle, and opted for specialized hunting of camelids as their main source of food (Aschero and Martínez, 2001). Table 1 summarizes the correlated changes in the environment and in human populations by period. These changes did not happen all at once across the region; the geography of the origins of animal domestication and social complexity can be best described as a mosaic pattern.

**Table 1.** Correlation between climate, environment, and main features of the archeological record for the human occupations for the Holocene

Period	Climate and environment	Features of human occupation
Early Holocene (12890–9200 cal. BP)	Stable, moist, and cold	First human settlement of the region
	Weak seasonality in precipitation	Small occupations
	Positive hidrological balance	Low artifact diversity
		Low transport rates of artifacts between localities
		Opportunistic use of animal resources (high diversity)
		Residential mobility
Middle Holocene I (9200–7100 cal. BP)	Arid and warm, marked seasonality in precipitation	More diversity of projectile points
	Environmental fragmentation	New hunting techniques with new weapon kits
	Negative hydrological balance	Grinding tools
	Shor-term climatic variations	Logistical mobility
	Long-term directional variation toward aridity	Specialization in animal use
Middle Holocene II (7100–3770 cal. BP)	Extreme regional aridity	Subsistence diversification (camelid domestication and introduction of cultivated plants)
	Negative hydrological balance	Social complexity
	Fragmentation with habitat loss	Reduction of mobility
	Short term incremental variation (first ENSO)	Appearance of the first villages at the end of the period
	Slightly more humid as from 4470 cal. BP	

Domestication is a process of interaction between an animal species and humans. Darwin (1868) made explicit that domestication includes the raising of animals in captivity that can occur without a conscious effort on the part of people and increases animal fertility, allowing them to have greater plasticity. According to Price (1984, 2002), domestication is an evolutionary process marked by the genotypic adaptation of animals to the captive environment. A domesticated animal is one whose mate selection is influenced by humans and whose docility and tolerance to humans are genetically determined.

The first step of the domestication process is based on the relationship of a phenotypically plastic species habituated to human presence, and occurs before any genotypic change; therefore, it can last a long time. This process, called the Baldwin effect, is an evolutionary transition from a facultative tolerance to humans toward a dependence on them; at this stage, the animal population becomes accustomed to the human presence and a selection mechanism for docility begins to function (Crispo, 2007; Francis, 2015).

For llama domestication, a multilocation model has been developed that includes two phases: herd protection and captivity-selective breeding (Yacobaccio and Vilá, 2016). Herd protection refers to human intervention in guanaco populations, or population subgroups, whose individuals are protected from its nonhuman predators and are facilitated access to feeding areas. A second step is the captivity and selective breeding of certain individuals. In the phase of herd protection, channeled by the Baldwin effect, people are a neutral stimulus. In the second step, when people become a positive stimulus—usually associated with the presence of food or shelter—an associative kind of learning emerges that goes beyond habituation and generates the taming process. Tameness is a condition for reproductive manipulation, as well as for the isolation of individuals in confinement or captivity. This step involves a greater degree of handling and isolation, meaning the existence of a physical barrier between wild population and captive herds. The space constraint increases animal density, resulting in changes in the social structure of the group of camelids and triggering genetic adaptation to captivity (Yacobaccio and Vilá, 2016: 10–11).

**Figure 2.**
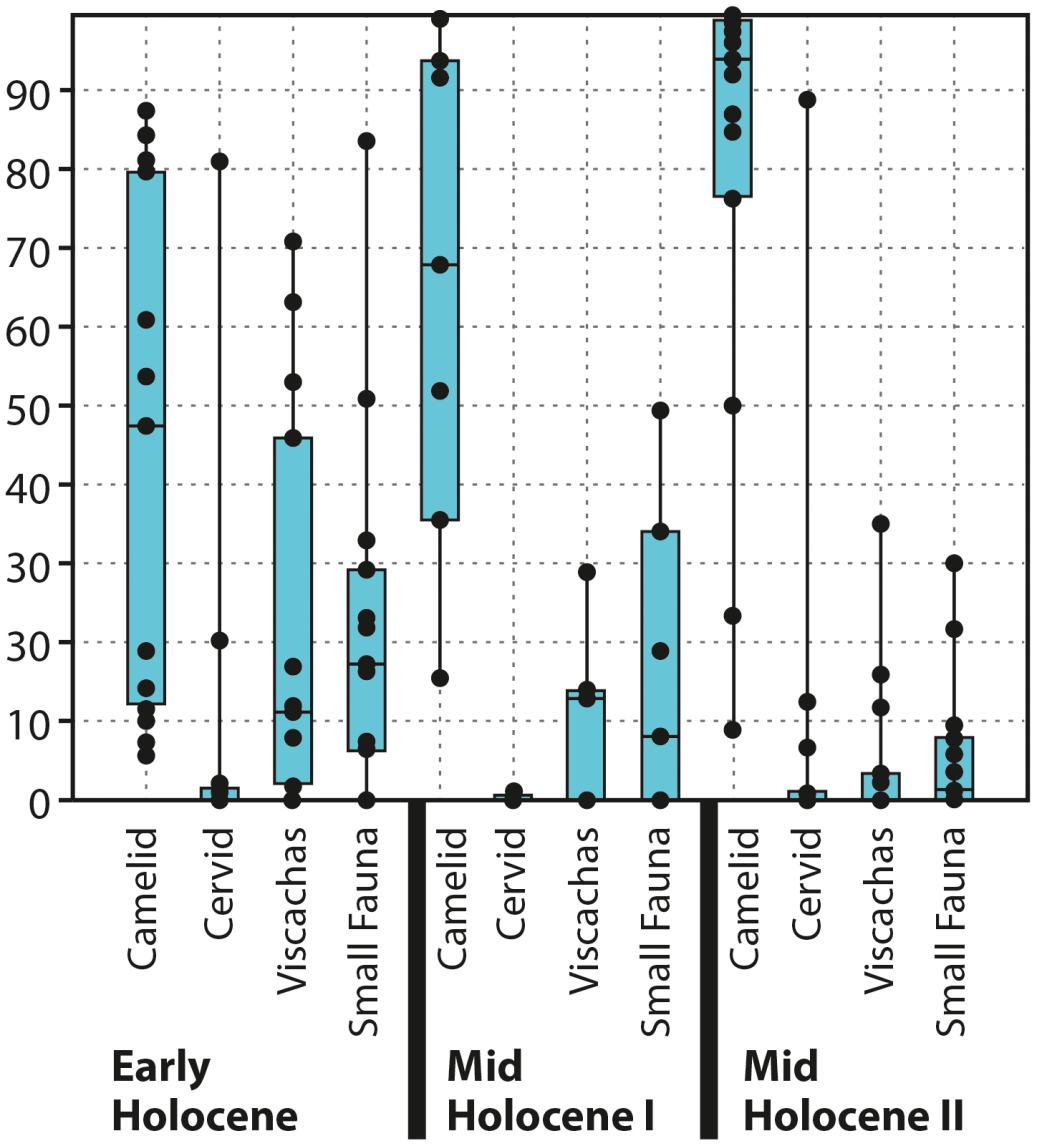
Use of animal resources during the Holocene in the high Andean region of Northwestern Argentina and northern Chile. Box and jitter graph of data represented in percent of identified bones from 28 archeological sites. See location of some of them in Figure 3.

Of the four pathways of domestication process, camelids are models for the “prey pathway,” which includes medium to large ungulates targeted as prey (Larson and Fuller, 2014). In the transition from game management to herd management, hunter-gatherers changed their hunting strategies to maximize the availability of the prey (Larson and Fuller, 2014). In the Southern Andes, this is suggested by several proxy data. The representation of camelid bone remains in archeofaunal assemblages increased through time from 29.7% to nearly 90%, whereas other taxa, generally small fauna, are markedly reduced (Figure 2).

The composition of zooarcheological assemblages and their temporal change is reflected in their diversity (Shannon *H*) and dominance (*D*) indices. The Shannon *H* index accounts for the abundance and evenness of species in an assemblage, and *D* is a measure of dominance that reveals the most conspicuous and abundant species. From the Early to the Mid-Holocene II (12890–3770 cal. BP), diversity decreased from 0.93 to 0.29, whereas *D* increased from 0.52 to 0.84 (Yacobaccio, 2013; Yacobaccio et al., 2017). These two measures can be used as proxies of generalized vs. specialized use of fauna, in this particular case, camelids. The most ancient radiocarbon dates associated to this change in diversity and dominance indices have a pooled mean of 6177 ± 39 cal. BP. This suggests that before that date, hunter-gatherers had a mostly generalized subsistence base on a regional scale in which different habitats were used according to gross species abundance, with the exception of patches that offered a limited range of resources in great quantities, like some rodent colonies or bird nesting places. After ca. 6200 cal. BP, a specialized economic system emerged and resilient habitats were used mainly to increase the use of the focal resource. The emergence of this economic system based on the intensification of the use of camelids coincided with the earliest evidence for herd protection (Yacobaccio and Vilá, 2016: 9).

Here we will review the evidence for the domestication of camelids in the Southern Andes arranged according to the steps of the model described above. I recommend a recent review of the evidence for the Central Andes by Moore (2016). However, in Discussion and Conclusion, I will compare some specific issues between both regions.

## Genetic Evidence

Genetic studies have shown that the two wild species belong to two distinct groups and are therefore good taxonomic genera. Vicuñas are differentiated into two clades; that is, they are two parts of a group that descend from a common ancestor, according to their subspecific assignment (the northern, *Vicugna vicugna mensalis* and the southern *Vicugna vicugna vicugna*). Guanacos can also be divided into two subspecies: the northern *L. g. cacsilensis* and the southern *L. g. guanicoe*, the latter having a greater distribution that includes the southern Andes and Patagonia (Kadwell et al., 2001; Marin et al., 2017). Llamas and guanacos form a monophyletic group in a clear antecessor-derived species process. Alpacas are associated with vicuñas from mitochondrial genome (Marín et al., 2017), and also associated with the guanaco lineage when microsatellites were analyzed (Kadwell et al., 2001). Microsatellites notwithstanding, these studies concluded that the llama was derived from the guanaco and the alpaca from the vicuña (Wheeler et al., 2006; Marin et al., 2017).

An analysis of genetic diversity in the hypervariable region of the mitochondrial genome in Bolivian llamas and alpacas published by Barreta et al. (2013) confirmed that guanacos are the ancestors of llamas, but the origin of alpacas remains unclear (Barreta et al., 2013). The article found exclusive haplotypes shared between alpacas and vicuñas, but a significant number of alpacas (51%–63% of the samples) were found to belong in the guanaco clade. This indicates a high degree of hybridization, suggesting that alpacas had a mixed origin, or alternatively, that an introgression occurred during or after domestication. If the first hypothesis were confirmed, it would provide strong proof that alpacas were domesticated after the llama. The mitochondrial control region indicates that all the haplotypes shared between guanacos and alpacas also exist in llamas. This could indicate that hybrids between domestic forms were common. The model derived from the results of Barreta et al.’s article lend support to the idea that alpacas resulted from interbreeding between vicuñas and llamas. Likewise, from the confirmation of the existence of two lineages of guanacos (northern and southern) and the finding that some llamas share haplotypes with southern guanacos, they conclude: “The present study would support also at the genetic level and taking into account the archaeological evidence, the existence of additional llama domestication centres in Argentina and Bolivia” (Barreta et al., 2013: 8). The hybridization produced since the 16th century AD, after the Spanish conquest of the Andes, produced mitochondrial lineages shared between different species, limiting the power of this line of inquiry to clarify the origins of domestic camelids. For this reason, it is of fundamental importance to carry out paleogenomic studies. Of the various studies carried out in South American camelids (Weinstock et al., 2009; Westbury et al., 2016; Díaz Marotto, 2018; Abbonna et al., 2020), we will comment here on Díaz Marotto (2018) for its relevance. The author analyzed samples from three archeological sites in the Salar de Atacama area (Chile) dated between 2750 and 2500 cal. BP. This work does not refer to the earliest moments of domestication, but rather to the moment when pastoralism became the predominant economic strategy among local human groups. Díaz Marotto studied the complete genome of 77 bone samples, obtaining conclusive evidence that llamas were domesticated derivates of guanacos. In this case, a distinction between subspecies of guanacos was made, confirming that both *L. g. guanicoe* and *L. g. cacsilensis* were the ancestors, in contrast with previous studies of current genomes (Marín et al., 2007) that had proposed *L. cacsilensis* as the only ancestor. In turn, the complete mitochondrial genome of alpacas shows that this species, also domesticated before the Spanish conquest, has a much closer relationship with the guanaco/llama lineage than with vicuñas. Díaz Marotto determined a clade of domestic species where llamas and alpacas are grouped very closely, suggesting that the llama was the first domesticated species, followed by the emergence of alpacas as a result of sustained interbreeding of female llamas with male vicuñas. Likewise, she agreed with Barreta et al. (2013) that the South-Central Andes was a domestication center independent from that of the Central Andes, based on the evidence of the domestication of the *L. g. guanicoe* subspecies. This conclusion is in line with more general arguments about the number of domestication events in ungulates. The use of genetic sequences has led numerous authors to conclude that animal domestication was a great deal more frequent and evenly distributed than previously thought. This claim is based “on the affinity between DNA sequences of domestic animals and their wild counterparts and the assumption that branching patterns on phylogenetic trees reflect independent domestication episodes. This rationale has been used to support claims for multiple and independent domestications of genetically and geographically divergent populations” (Larson and Fuller, 2014: 214). This argument takes into consideration pigs, goats, sheep, horses, cows, and now, we may add, llamas.

## Main Archeological Evidence

Osteometry has been used as a proxy to study the domestication process by detecting changes in the size of individuals. Indeed, South American camelids have a size gradient from smallest to largest: vicuña—alpaca—northern guanaco—llama. This defines two groups: the small one—vicuñas and alpacas—and the large one—guanacos and llamas. As can be seen, both groups contain wild and domestic camelids. In the large group, there is a complicated fact, and it is the variability of the guanaco size; *L. guanicoe* has a high clinal dispersion, which influences its size. Patagonian or southern guanacos are much larger than northern or North Andean ones. That is why we have to be very careful with the reference measurements used to compare with archeological samples. These issues have been widely debated in Andean zooarcheology (Izeta, 2008; Cartajena, 2009; Gasco et al., 2014; Hernández and L’Heureux, 2019). In the large group, the variation in guanaco size imposes certain restrictions on the determination of species (guanaco vs. llama) based on osteometry alone. There is a “zone of uncertainty” defined by an overlap in size between small llamas and North Andean guanacos. However, the larger measurements beyond this overlap zone can be determined as *L. glama* without a doubt.

As far as we know today, the first osteological evidence of a change in size in camelids that could reveal the modifications produced by a domestication process made its first appearance in the Southern Andes at approximately 7100 yr cal. BP. This evidence is an increase in the width of the distal metacarpus, along with an increase in the size and robustness of other bones, such as phalanges, scapulae, and humerus. These specimens are larger than the known sizes for North Andean guanacos, and are comparable to modern llamas, or even larger (Cartajena et al., 2007). This points to the emergence of a camelid similar in size to the largest among current llamas. In the herd protection phase (7100–4500 cal. BP), there was an increase in size variability, especially with the emergence of very large specimens that were first detected in the archeological record at the Hornillos 2 site (layer 2), but later increased their distribution significantly between 5800 and 4200 cal. AP, when their presence was noted in numerous archeological sites in the region (Izeta, 2010, Figure 3).

**Figure 3.**
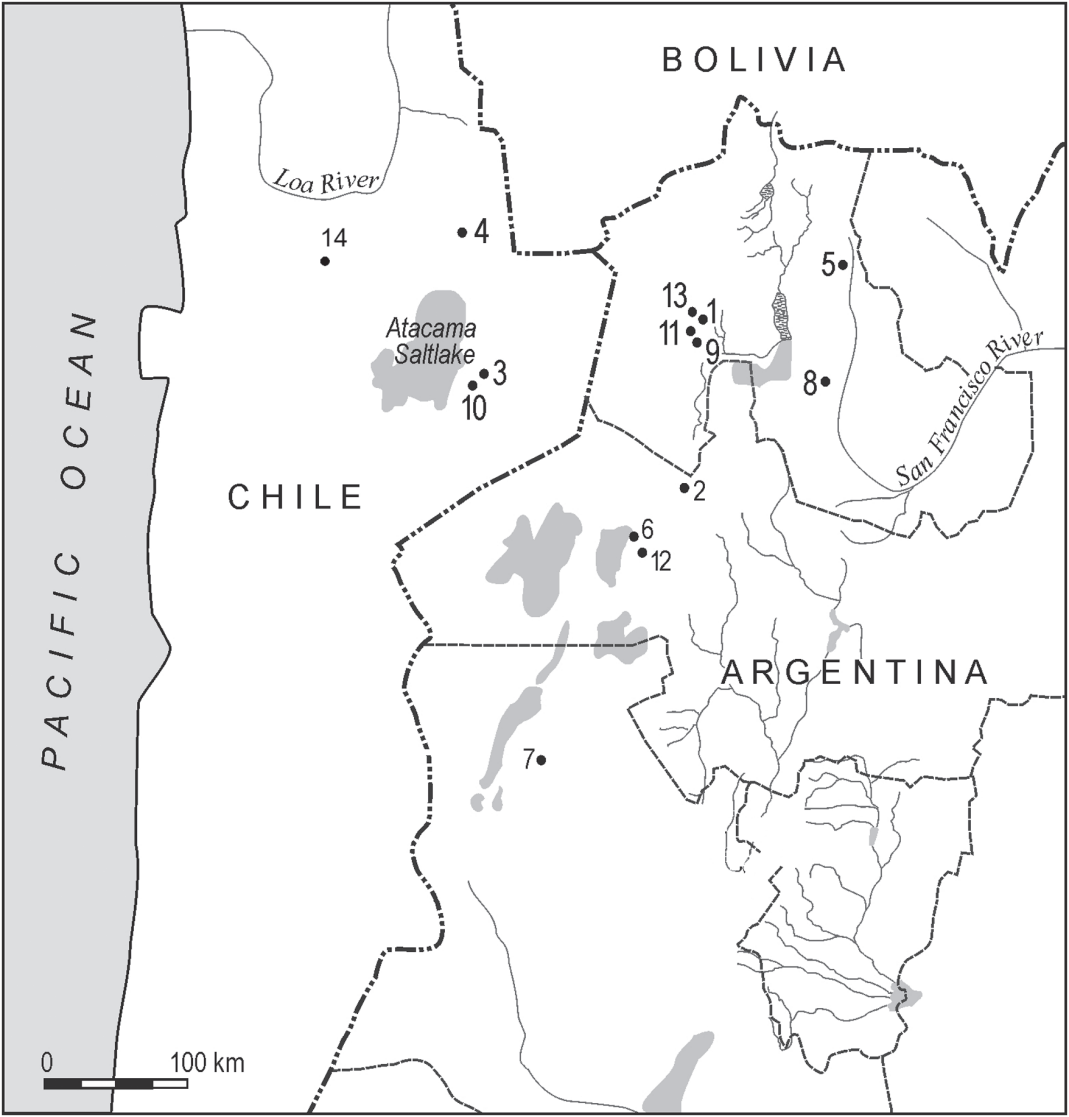
Map showing selected sites with camelid domestication evidence. 1. Hornillos 2; 2. Alero Cuevas; 3. Tulán 52; 4. Puripica 1; 5. Inca Cueva 7; 6. Pozo Cavado; 7. Alero Sin Cabeza; 8. Huachichocana III; 9. Alero Unquillar; 10. Tulán 54; 11. Cueva Quispe; 12. Casa Chávez Montículos; 13. Huirunpure; 14. Topater 1.

**Figure 4.**
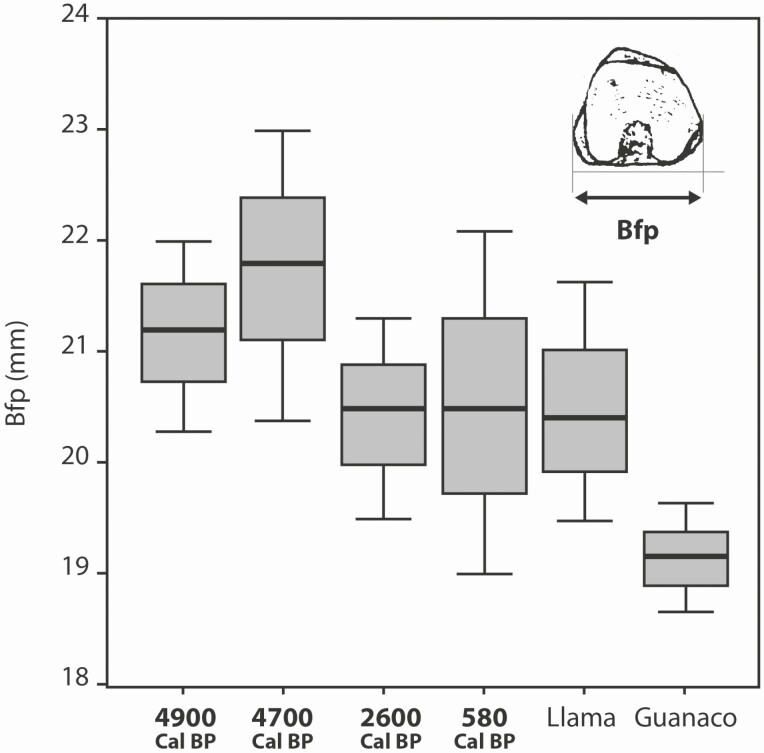
Measurement of the breadth of the phalanx I facies articularis proximalis (Bfp) through time. Between 4900 and 4700 cal. BP, larger animals than today llamas are noted. Later, the sizes are the same as the current llamas and bigger than northern guanacos.

The variation in size that we refer to here is summarized in Figure 4. From the variation in breadth of the phalanx I facies articularis proximalis, it can be inferred that between 4900 and 4700 cal. BP, there was a significant proportion of camelids that were larger than current llamas, followed by a reduction in size by 2600 cal. BP, setting the median and interquartile range equal to that of current llamas. From that date on, bone remains equivalent to the size of current llamas were found in other ecosystems, such as mesothermal valleys (between 1500 and 2900 masl) and the lowlands of the Chaco, in numerous archeological sites (Izeta, 2010, Mercolli, 2019, Del Papa, 2020). At 580 cal. BP, during the Inka period, the size of llamas completely coincides with that of today.

Harbers et al. (2020) found that “mobility reduction induces a plastic response beyond the shape variation of wild boars in their natural habitat, associated with a reduction in the range of locomotor behaviours and muscle loads” and that produces changes in the calcaneus shape due to captivity. This is extremely interesting because identifies osteological modification as a consequence of confinement. The case of llamas seems to have been different because there was no such a reduction in mobility on that scale. Moreover, as llamas were used as pack animals, their mobility remained to be high. However, the zooarcheological record shows a reduction of size variability after pens appeared as can be seen in Figure 4, where size become stabilized since 2600 cal. BP. Recent research on morphometric analysis made on guanaco and llamas first phalanges are promising, but no conclusive yet because the two species have not been differentiated in these analyses (Hernández and L’Hereux, 2019).

Other paleoenvironmental and archeological evidence accompanied the change in size of the camelids and point toward a change in the camelid-human relationship. Pollen analysis in several localities of the Puna has identified a greater abundance of Chenopodiaceae–Amaranthaceae and *Pennisetum* from ca. 5300 to 4869 cal. BP, which is consistent with more intensive human impact on wetlands due to environmental management practices, perhaps including periodic burning to increase patch productivity.

This change in the human–camelid relationship is also seen in the appearance of bone pathologies. Cartajena et al. (2007) recorded periostitis in distal phalanges and metapodia from the Tulán 52 (ca. 4900 cal. BP), and Puripica 1 (ca. 4700 cal. BP) sites. These exostoses are due to the proliferation of the bone, possibly caused by long-term irritation of the periosteum. They also observed osteophytes produced by arthropathies due to the living conditions of the animals, perhaps related to the long periods of exercise that are characteristic of pack animals. Labarca Encina and Gallardo (2015) analyzed 14 bones with pathologies from the Topater 1 cemetery (Calama, Chile) dated between 2517 and 1868 cal. BP. Most of the pathologies occur in limb bones that, due to their size, have been assigned to *L. glama*. At this site, most of the phalanges show exostoses, an abnormal formation of new tissue on the outside of the bone. Its manifestation is mild to moderate and is located in the diaphysis and, to a lesser extent, on the dorsal and lateral faces of the epiphyses. These pathologies were attributed to constant trauma to the joints and could result from environmental factors, such as walking on uneven ground, as well as the excessive use of the animal due to cultural practices, as would be the case of pack animals. A llama head with articulated vertebrae recovered from an inhumation at the Huachichocana III site (3170 and 2867 cal. BP, Figure 3) also provided information on bone pathology. The cervical vertebra C2 (axis) presents periostitis due to direct trauma caused by a tie rope or due to an excessive use of this articular section of the neck. The use of a muzzle to restrain the animal is the most probable explanation for this pathology. In addition, the analysis of stable carbon and nitrogen isotopes reinforces the idea that this specimen’s diet was strongly determined by human intervention (Yacobaccio et al., 2018).

The use of muzzles at such an early time would not be uncommon, as ropes had already begun to appear in the archeological record. These ropes can be interpreted as a technological innovation related to the onset of pastoralism. Indeed, at Alero Unquillar (Figure 3), in which two metapodia were determined to belong to a llama by osteometry, a rope made with local grasses dated at 3989–3570 cal. BP was recovered. Likewise, the grave goods of human burial number 4 in the Huachichocana III site included remains of ropes made with palm leaf fibers and local bromeliads (*Tillandsia usneoides* or *Deuterocohnia*; Lema, 2017). This burial is relatively contemporary with burial 3—the one containing the llama head—and it could be associated with two radiocarbon dates from ca. 3360 to 3170 cal. BP. The appearance of ropes has direct implications for the development of gripping or restraining technology associated with herd management.

**Figure 5.**
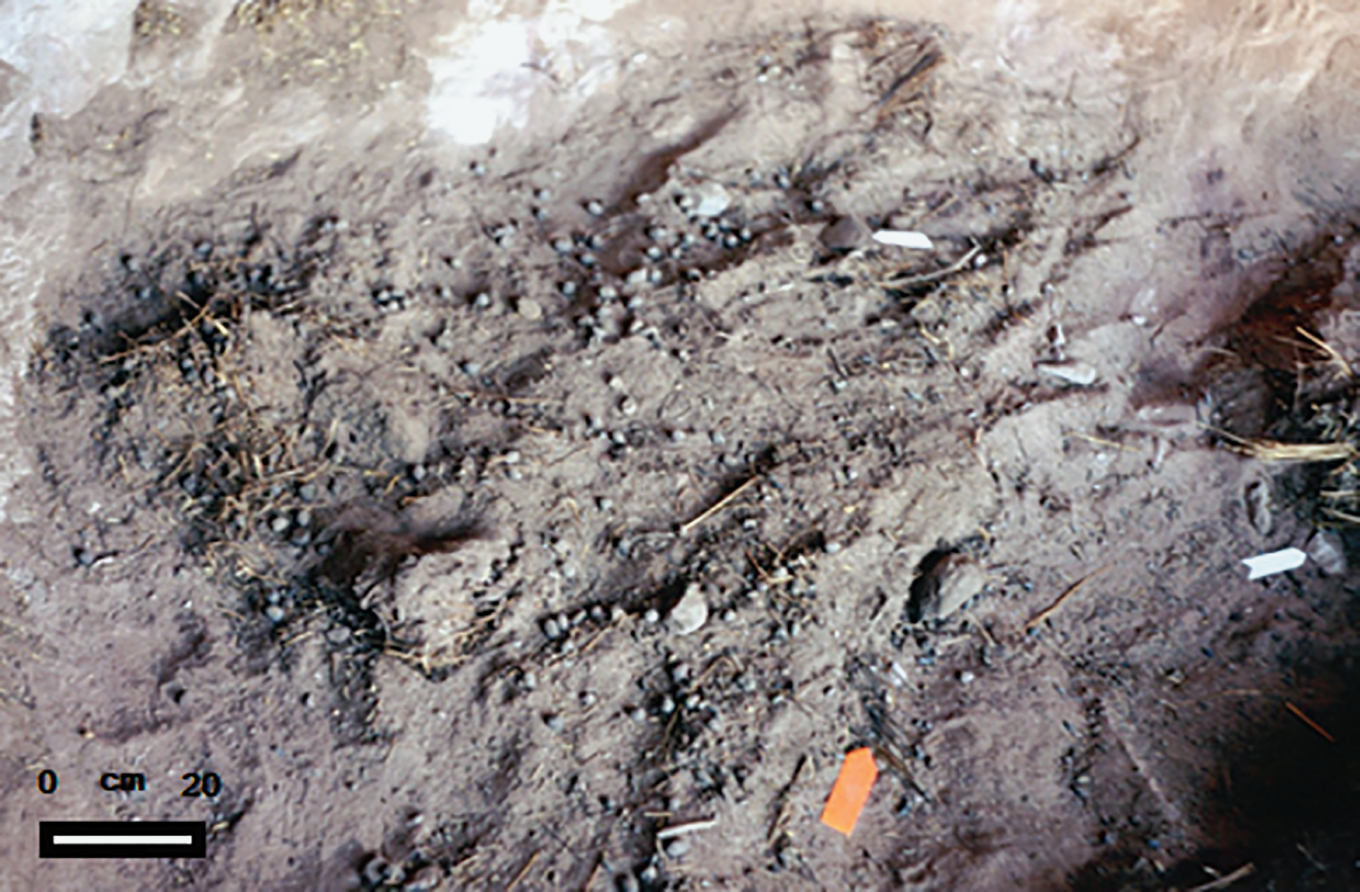
Layer of feces (guano) on the ground of Inca Cueva 7, dated in 4635 and 4232 cal. BP. This is the most ancient evidence of pens from Northwestern Argentina.

A change in phase of the domestication process occurred with the emergence of confinement technology, that is, pens. The first corrals were small caves, such as Inca Cave 7, whose entrance was covered by a wall of stone boulders. Given its size, it was probably used to keep young camelids in confinement. A layer of guano that covered the ground within the cave was helped to determine its function as a corral (Figure 5).

As mentioned above, two metapodia determined as belonging to very large llamas were recovered from this site. The corralling episode has been dated between 4635 and 4232 cal. BP. In the Tulán gorge, Cartajena et al. (2007: 168) observed that the “high and rocky slopes are used as natural boundaries on both borders; on softer slopes big regular boulders have been arranged to enclose pens. Boundary lines that cross the ravine are interrupted in the stream part to avoid holding back the water; palisades or similar solutions were probably used to bound areas close to the stream, allowing the enclosure of pens.[…] Pen dimensions (ca. 300 × 80 m) suggest a considerable amount of labour.” This corral can be dated at around 2600 cal. BP.

## Discussion and Conclusion

The transition from hunting to herding has been a complex one. Herd protection involved changes in camelid behavior, but also modifications in the human strategies used to approach wild camelids. This phase lasted for a long time, from 7100 to 4500 cal. BP, when corrals made their first appearance in the region.

Environmental fragmentation promoted the aggregation of humans and wildlife in resilient habitats, such as wetlands, thus creating the conditions for the development of a closer and more stable relationship between people and camelids. Then, as a condition for herd protecting and habituation, human communities reduced their mobility, stabilizing their residence in these areas where grazing resources were more concentrated. The entire time span characterized by herd protection also was accompanied by an increase in human population. The archeological sites show evidence of more intensive occupation at this time, and about 4900 cal. BP, the first site with several stone enclosures could have functioned as places where human populations gathered periodically (Núñez and Perlès, 2018).

Moreover, the grouping of radiocarbon dates from archeological sites in the region has been analyzed as indicators of human demography or anthropogenic signal in the Holocene (Muscio and López, 2016). This study suggests that after 6177 cal. BP, the anthropogenic signal increased, reaching its maximum at 4700 cal. BP. This date correlates quite well with the appearance of corrals or courtyards in the archeological record, which points toward an intensification of the camelid domestication process. This is a significant finding because, for the diffusion of an innovation to take place—in this case, domesticated animals—there need to be long-reaching interaction networks sustained by large, interconnected populations. Evidence of such interaction networks can be provided by the existence of domestic llamas in the microthermal valleys and lowlands of the Chaco region since at least 2100 cal. BP, which reveals a relatively rapid dispersion of this innovation.

This process is contemporary with what happened in the Central Andes, in which early domestication indicators in the Puna de Junín occurred between 5470 and 3480 cal. BP (Moore, 2016). In southern Peru, in the Osmore Valley, evidence of domestication appeared between 4090 and 3677 cal. BP (Aldenderfer, 1998).

Taking the Andes as a whole, the knowledge that we have about the domestication of camelids is still very fragmentary, since there are portions of territory with no archeological evidence on the period that concerns us. Likewise, more genetic studies on ancient materials are needed, although according to what we know today, llamas would have been domesticated first and later, alpacas, which resulted from introgressions of llamas with vicuñas (Barreta et al., 2013; Díaz Marotto, 2018).

There are still many unresolved issues and unanswered questions concerning the domestication of South American camelids. The next few years will hold many new and exciting insights for researchers in this challenging field.


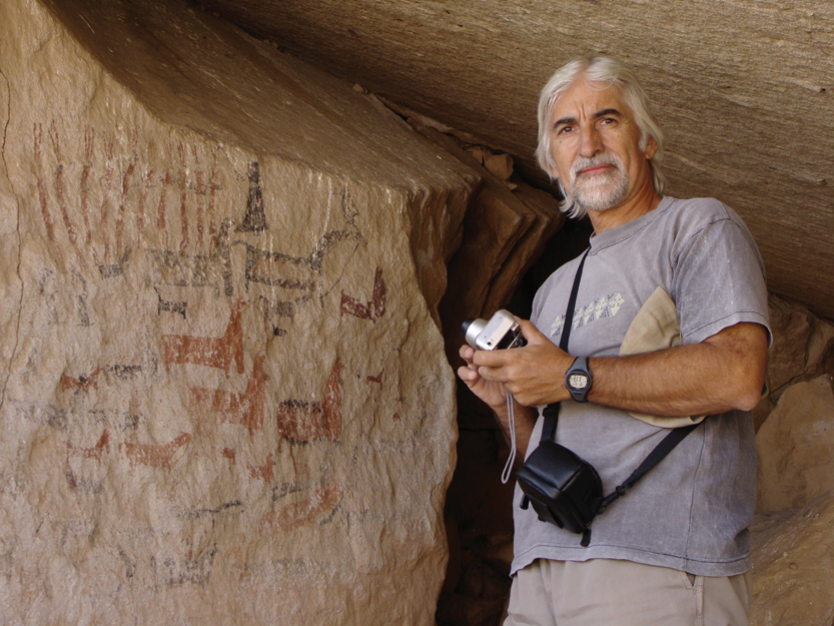


## About the Authors

**Hugo Yacobaccio** has obtained PhD from the University of Buenos Aires. Currently, he is superior researcher of the National Council of Investigation (CONICET), full professor of Archaeological Theory at the University of Buenos Aires, and member of the doctoral teaching committee at the University of Tucuman. His fields of interest are related to environmental archeology, archeozoology, animal domestication, ethnoarcheology, and the peopling of the South Andean Altiplano. Recently, he has been working on climatic change and its effects on pre-Hispanic society of the southern High Andes and on frames of reference for studying climatic change in the past. He also has been involved in projects concerning vicuña use and conservation and, signing several agreements with aboriginal communities of the Altiplano, in order to sistematize, protect, and create an interpretation center for the rock art (now under construction) in Barrancas (Puna of Jujuy Province, Argentina).
